# A tensor decomposition reveals ageing-induced differences in muscle and grip-load force couplings during object lifting

**DOI:** 10.1038/s41598-024-62768-8

**Published:** 2024-06-17

**Authors:** Chang Ye, Seyed Saman Saboksayr, William Shaw, Rachel O. Coats, Sarah L. Astill, Gonzalo Mateos, Ioannis Delis

**Affiliations:** 1 Department of Electrical and Computer Engineering, University of Rochester, Rochester, 14620 USA; 2 School of Biomedical Sciences, University of Leeds, Leeds, LS2 9JT UK; 3 School of Psychology, University of Leeds, Leeds, LS2 9JT UK

**Keywords:** Motor control, Data mining

## Abstract

Do motor patterns of object lifting movements change as a result of ageing? Here we propose a methodology for the characterization of these motor patterns across individuals of different age groups. Specifically, we employ a bimanual grasp-lift-replace protocol with younger and older adults and combine measurements of muscle activity with grip and load forces to provide a window into the motor strategies supporting effective object lifts. We introduce a tensor decomposition to identify patterns of muscle activity and grip-load force ratios while also characterizing their temporal profiles and relative activation across object weights and participants of different age groups. We then probe age-induced changes in these components. A classification analysis reveals three motor components that are differentially recruited between the two age groups. Linear regression analyses further show that advanced age and poorer manual dexterity can be predicted by the coupled activation of forearm and hand muscles which is associated with high levels of grip force. Our findings suggest that ageing may induce stronger muscle couplings in distal aspects of the upper limbs, and a less economic grasping strategy to overcome age-related decline in manual dexterity.

## Introduction

With advanced age comes decline in motor function^52^. An important daily-life skill required for older adults (OA) is the ability to lift objects^10,43,54^. Several daily tasks, such as carrying heavy objects, require coordination of the two hands (bimanual)^63^. However, the majority of research examining grasping in OA has focused on unimanual control^11^ despite the ecological validity of bimanual object manipulation^40^ and its growing use for therapy or rehabilitation (e.g., in stroke^35,41^).

In unimanual settings, OA have been shown to be slower during the pre-loading and loading phases of the lift^10^ and exert higher levels of grip force as well as a larger safety margin when lifting objects^10,12,23^. These findings indicate that ageing induces differences in grasping strategies^25^. However, a characterization of the motor patterns underlying these changes at the kinetic and muscle activation levels is currently missing. A potential explanation for this gap in the literature is the lack of a computational approach that enables characterizing the dynamic interaction between arm and hand muscle activations with grip and load force during grasp-lift-replace tasks^26,36^. As a result, even when differences in force variables are observed as a result of ageing, the corresponding differences in muscle activity remain largely unknown^49^.

**Figure 1:**
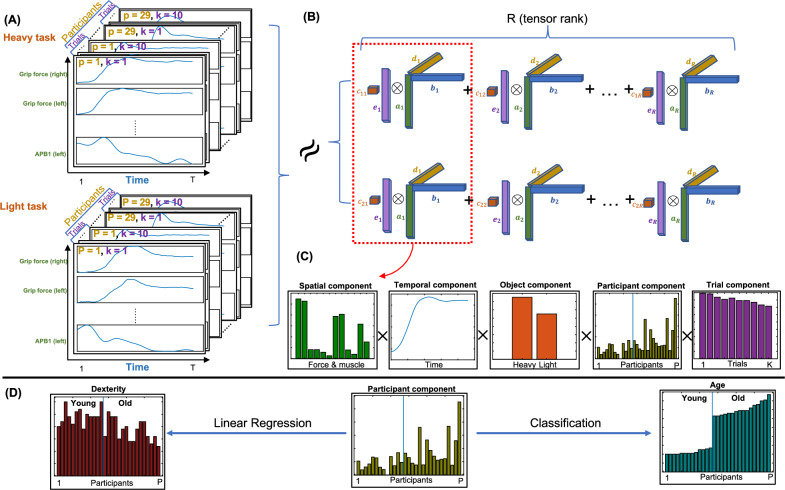
A schematic illustration of the tensor data analysis pipeline implemented in this study. (**A**) The 5-way tensor data $\underline{{\textbf{X}}}$ (Spatial$\times$Temporal$\times$Object$\times$Participants$\times$Trials); (**B**) The NCP decomposition $\underline{{\textbf{X}}}\sim {\textbf{A}}\circ {\textbf{B}}\circ {\textbf{C}}\circ {\textbf{D}}\circ {\textbf{E}}$; (**C**) The first mode of the estimated factor matrices including a spatial (muscle and force) component, a temporal component, an object (heavy or light) component, a participant component and a trial component; (**D**) Using the participant component to predict clinical test values (e.g., manual dexterity) or participant characteristics (e.g., age) using regression and classification analyses.

Thus, in this study we investigate how advanced age influences muscle couplings as well as the associated motor strategies during object lifting^42^. To assess the effect of ageing, both younger and older adults (YA and OA respectively) performed a bimanual grasp-lift-replace motor task. The combined recordings of muscle activity as well as grip and load forces can be represented as 5-way arrays, or tensors ^37,55^, indexed by space (muscles and forces), time, lifted objects (heavy and light), participants (including both YA and OA), and trials (multiple repetitions for each object); see also Figure 1(A). In order to analyze the aforementioned data and unveil the main patterns describing the lifting movements across participants, we introduce a high-order (order 5 here) tensor decomposition approach based on non-negative canonical polyadic (NCP, also known as non-negative CANDECOMP/PARAFAC) tensor decomposition ^39^; see Figure 1(B). The non-negativity constraint of this method yields a parts-based representation of the motor signals^46^. Crucially, since muscle activations and forces cannot take negative values, the extracted 5-mode components are directly interpretable as muscle activation and force patterns (mode 1) with their corresponding temporal profiles (mode 2) and also specific level of recruitment for each object (mode 3), participant (mode 4) and trial (mode 5); see Figure 1(C) for an example and^16,24,58^ for related approaches with three modes.

All in all, here we ask if motor and kinetic patterns of object lifting movements change as a result of ageing. We thus use this tensor analysis methodology to uncover these changes, characterize the functional roles of the identified patterns, and relate them to manual dexterity differences across individuals; see Figure 1(D). Importantly, muscles and forces are tightly linked and including them both in such a decomposition framework enables not only the extraction of muscle synergy patterns, but also revealing their relationship with the forces they are associated with in task space ^1,2^. Relating muscle synergies to functional outcomes is a crucial element of analysis often lacking in the muscle synergy literature (but see e.g.^3,5,47,57^). The proposed approach, as well as other recent ones ^51^, can fill this gap by offering a direct mapping between muscle synergies and motor outputs at the kinetic or kinematic level ^4^.

**Figure 2:**
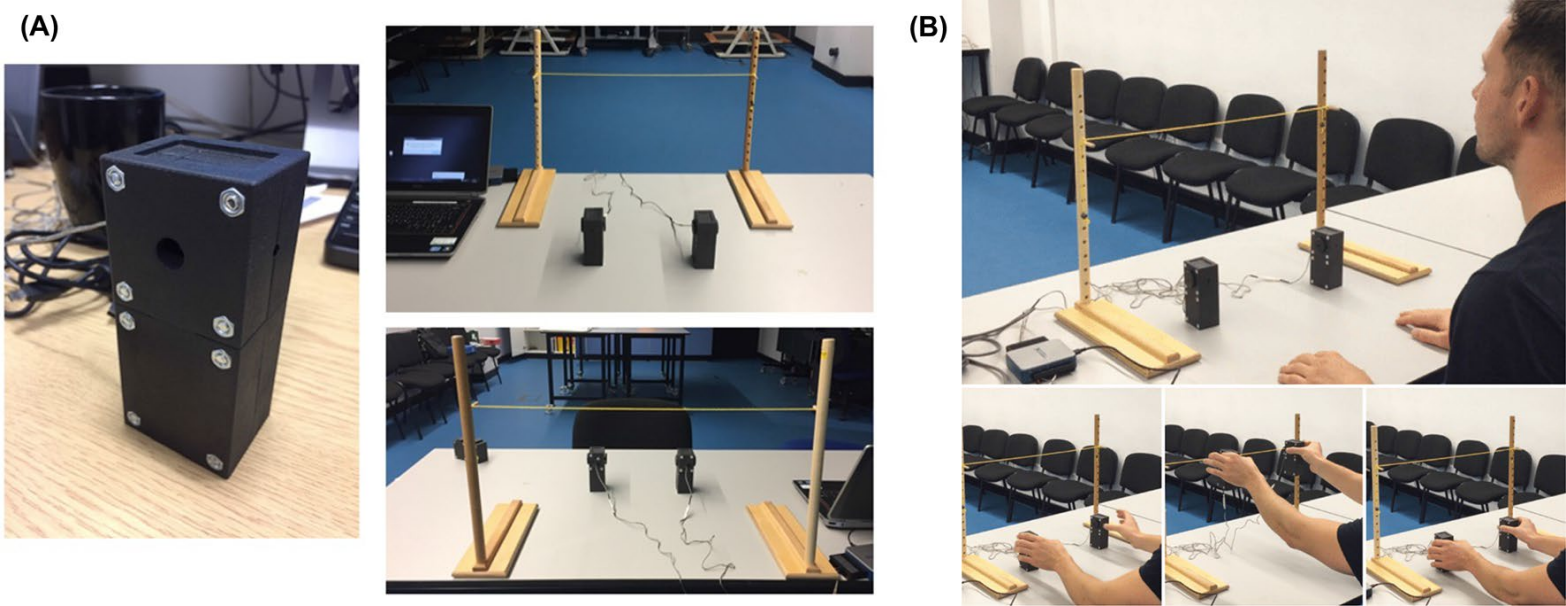
(**A**) Left - manipulandum body. Right - the final manipulanda setup for testing, from the participant’s view (upper) and face-on to where the participant would be sat (lower). (**B**) The phases of the grasp-lift-replace task - top: start position for each trial, bottom left: the initial grasp of the manipulanda, bottom middle: manipulanda raised to target height, bottom right: manipulanda replaced back on their starting locations.

**Table 1:** Indices for the spatial components (mode 1) with descriptions of the measured kinetic variables and EMG-based muscular activity across the two upper limbs.

Index	Force	Index	Muscle	Index	Muscle
1	Grip force (right)	5	Anterior deltoid (right)	9	Flexor Carpi Radialis (right)
2	Grip force (left)	6	Anterior deltoid (left)	10	Flexor Carpi Radialis (left)
3	Load force (right)	7	Extensor carpi radialis (right)	11	Abductor Pollicis Brevis (right)
4	Load force (left)	8	Extensor carpi radialis (left)	12	Abductor Pollicis Brevis (left)

## Results

### Overview of the experimental task and analyses conducted

**Figure 3:**
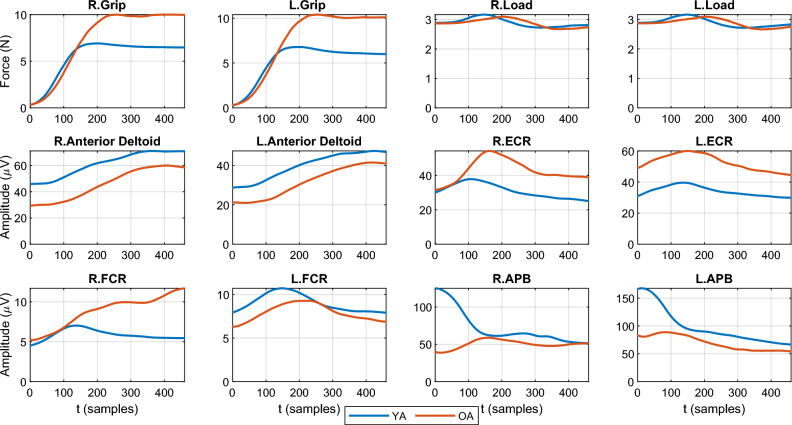
The group (YA and OA), object type (H and L), and trial averaged time courses for each of the recorded kinetic variables and EMG signals of four muscles in both arms. While on average differences between YAs and OAs are apparent, from these time series one a) cannot quantify to what extent these differences are consistent across individuals; and b) what combinations of muscles and/or forces are consistently different (or similar) between OAs and YAs.

We employ a bimanual grasp-lift-replace protocol to evaluate cross-limb coordination during the lift phase and combine it with EMG measurements of muscle activity across the two upper limbs to a) quantify the motor signals that contribute to the generation of these forces; and b) provide a window into the motor strategies used to perform an effective object lifting movement^50^. The $P = 29$ participants (13 YA and 16 OA) were asked to bimanually grasp and lift heavy (H) or light (L) objects; see also Figure 2 (A) for a depiction of the manipulanda and Figure 2 (B) for the phases of the grasp-lift-replace task conducted. Throughout, the EMG signals of four muscles of both arms and hands, namely, Anterior Deltoid, Flexor Carpi Radialis (FCR), Extensor Carpi Radialis (ECR) and Abductor Pollicis Brevis (APB); as well as kinetic variables, namely the Grip Force and Load Force, were recorded on each upper limb (left, right) as temporal sequences. Table 1 summarizes the muscular activity and kinetic variables measured. Each participant performed $K = 10$ repetitions for each object grasp and lift. An advantage of this experiment was the simultaneous recording of kinetic variables and muscle activations across the two limbs (see Figure 3 for the recorded group, object type, and trial averaged signals), which enabled the study of the relationship between the generated forces and the corresponding muscle signals over time.

To this end, we firstly fed the EMG and force data recorded during lifting movements to a NCP decomposition algorithm to obtain a concise spatial and temporal characterisation of the main motor and kinetic patterns across participants and objects. Inspection of Figure 3 reveals on average differences between YAs and OAs, but the proposed NCP approach can identify distinct (or shared) age-related patterns between OAs and YAs, while at the same time dissecting how much is each component recruited by each participant and on each trial. Secondly, to investigate the relationship between age and manual dexterity with the activation of each component, we applied linear classification and regression analyses. We first used the estimated participant-mode components (representing component recruitment for each participant) to predict which age group, OA or YA, each participant belongs to. We then employed linear regression and correlation analyses to quantify the contribution of each component to the prediction of age and manual dexterity.

**Figure 4:**
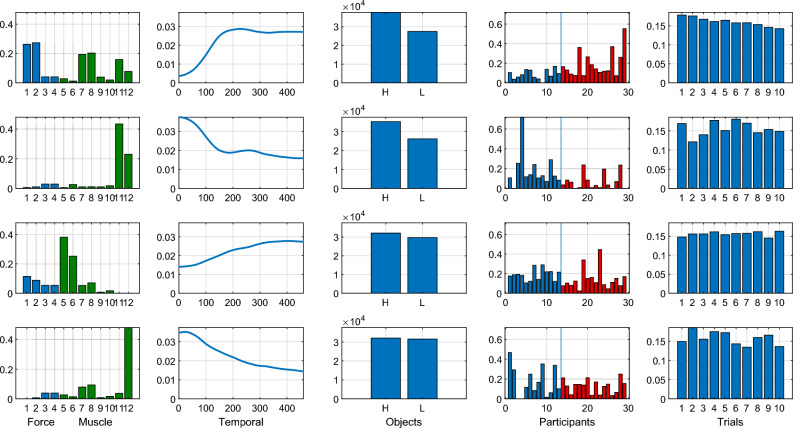
The estimated factors for the 5-way tensor with $R=4$ components (in rows). The first column shows the decomposition of the spatial mode ($M=12$) into $R=4$ different components, where the forces and muscles are color coded (blue and green, respectively) for improved visualization. Indexing of forces and muscles is done as described in Table 1. The second column shows the four temporal ($T=460$) components and the third column shows the estimated object ($J=2$) factors. The fourth column shows the participant ($P=29$) factors, and the vertical line indicates the boundary of the young group (YA, left hand side, blue) and old group (OA, right hand side, red). The last column shows the four trial ($K=10$) components. Throughout, the magnitudes represent how much each variable (or trial) contributes to each component.

### Unveiling space-time-object-participant patterns of muscle activity via tensor decomposition

After data pre-processing (see Materials and Methods for details), we obtained the EMG data during the dynamic phase of object lifting (from the point of first contact with the object to the end of the lift) as a 5-way tensor $\underline{{\textbf{X}}}^{(5)}\in \mathbb {R}_+^{ M\times T\times J\times P\times K}$, where $T = 460$ is the length of the pre-processed signal sequence; $M = 12$ is the spatial dimension after concatenation of the four forces (Right and Left Grip and Load Forces) and eight EMG signals (from the Right and Left Anterior Deltoid, ECR, FCR and APB muscles) – ordered using the indexing shown in Table 1; $J = 2$ accounts for the heavy ($j=1$) and light ($j=2$) objects.

In order to extract the spatial and temporal components (or factors, both terms will be henceforth used interchangeably), one natural approach is to implement the non-negative CP (NCP) decomposition to the 5-way tensor directly and obtain components in all 5 modes, i.e., spatial, temporal, object, participant, and trial. Figure 4 shows the estimated components, where the magnitudes represent how much each variable (or trial) contributes to each component. The decomposition entails $R=4$ components, as determined according to a Variance Accounted For (VAF) criterion (see Materials and Methods for details).

The first component ($r=1$) represents a high grip to load force ratio coupled with bimanual synergies between the extensor carpi radialis longus (ECR) and the abductor pollicis brevis (APB) muscles which synergistically contribute to hand stabilisation during object lifting. The participant mode of this factor suggests a significant increase of the activation of this component with age ($p<0.05/4$, independent t-test with Bonferroni correction) and the object mode shows recruitment for both object weights with slightly higher activation for the heavy object. The trial mode of this factor also shows a significant ($p<0.0001$, Mann-Kendall test) negative slope of $-0.0037$ over trials. The second component ($r=2$) couples (a low grip to load force ratio) with high activity of abductor pollicis brevis (primarily on the weaker left hand) and shows a decreasing activation over lifting time with a peak at grasp onset and a slight decreasing trend with age. The third component ($r=3$) captures a bimanual activation of anterior deltoids with a high grip-to-load force ratio. The fourth component ($r=4$) represents a decreasing over lifting time co-activation of ECR and APB muscles coupled with a low grip to load force ratio. This component appears complementary to the first component with a slightly higher activation in YAs, which is not statistically significant ($p=0.39$).

### Classifying young versus old adults from the identified participant factors

Having identified the main components of grasp-to-lift movements, we then sought to characterise their functional roles by testing how well they predict differences across individuals. We first aimed to understand if their recruitment is influenced by the age of the participants. We thus performed a *classification* analysis aiming to predict the age group (OA or YA) of the participant from the participant mode factors.

**Figure 5:**
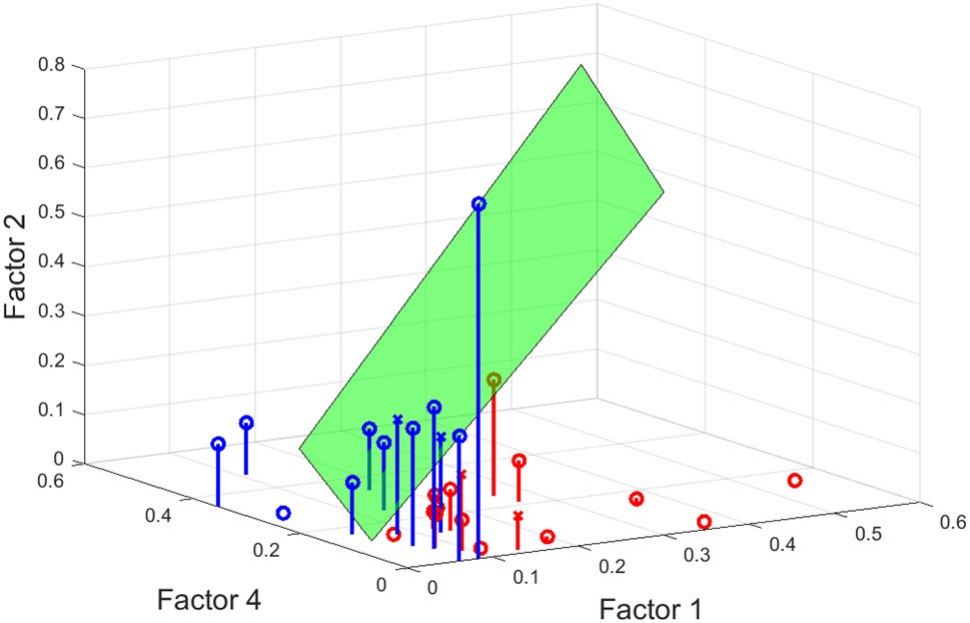
Age group (i.e. YA vs OA) classification using the first three participant-mode factors ($r=1,r=2$ and $r=4$) of the 5-way tensor as predictors. The green plane indicates the decision boundary of the linear classifier. The stem plots help visualize the points that lie on the different half-spaces defined by the decision boundary, and hence are assigned different predicted labels by the classification rule. Indeed, blue and red stand for the predicted labels (blue for YA, red for OA). The circle and cross indicate whether the prediction is correct (circle for correct prediction, cross for wrong prediction). The AUC and classification accuracy are 0.83 and 0.79, respectively.

To identify the most age-discriminating components, we used the participant factors as predictors of age group. We found that the highest performance based on our two performance measures (area under ROC curve – AUC and classification accuracy – Acc; see Materials and Methods for details) was ($\text {AUC}=0.83, \text {Acc}=0.79$) and was achieved by the combination of the first, second and fourth components (Figure 5), suggesting that age group can be best predicted by the joint recruitment of three factors ($r=1,r=2$ and $r=4$). For further reference, the performance attained by classifiers that use individual participant factors as predictors is: ($\text {AUC}=0.76, \text {Acc}=0.72$) for $r=1$; ($\text {AUC}=0.77, \text {Acc}=0.69$) for $r=2$; ($\text {AUC}=0.77, \text {Acc}=0.69$) for $r=3$; and ($\text {AUC}=0.49, \text {Acc}=0.66$) for $r=4$. The significant added value to classification performance of including all of these three components was also assessed via a permutation test described under Materials and Methods. Indeed, we found that the actual classification performance reported was higher than the 98, 96 and 91 percentiles (for factor $r=1$, 2 and 4, respectively) of the empirical distributions obtained for classifiers with randomly permuted individual participant factors, corroborating that all three components contribute considerably to this age group classification.

In particular, we observed higher (lower) activation of the first component ($r=1$) for OAs (YAs) and higher (lower) activation of the third and fourth components ($r=3, r=4$) for YAs (OAs). This suggests that, although both components were used by all participants regardless of age, their recruitment was dependent on the age group.

### Predicting age and manual dexterity from the identified participant factors

**Figure 6:**
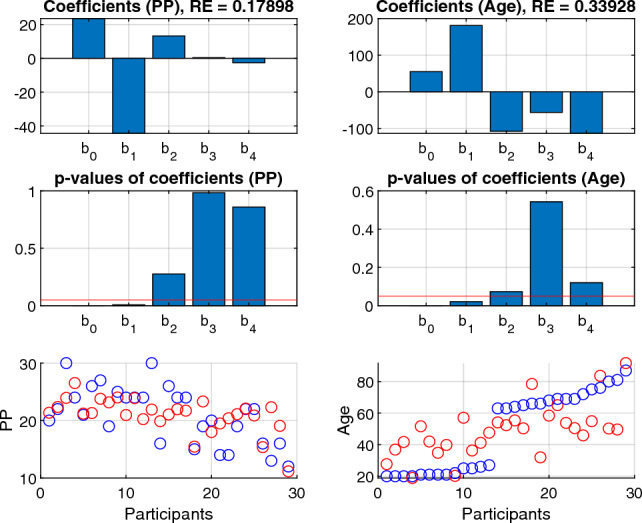
Results of linear regression using the participant modes of the 5-way model as predictors and *PP* or age as response. The first row shows the regression coefficients, where $b_0$ is the intercept and $b_1,...,b_4$ are the regression coefficients for the corresponding participant factors. The second row shows the p-value of the regression coefficients, with the red line indicating significant level 0.05. The third row shows the true and predicted *PP* (left) or age (right) for different participants. The blue and red points stand for the true and predicted values of *PP* and age, respectively.

We then asked if the estimated participant factors were predictive of the age and manual dexterity of the participant. Manual dexterity was assessed using the Purdue Pegboard (PP) test ^33^, which is the most widely used procedure for assessing hand function during therapy, rehabilitation, and for research purposes (more under Materials and Methods). Participants performed the test bimanually to obtain an overall measure of their manual dexterity, which we collect in a $P=29$-dimensional vector *PP*. We thus considered a *linear regression* model viewing the participant components as the predictors and the age or dexterity vector *PP* as the response. The estimated coefficients of the linear regression model, the p-values of the coefficients and the predictions for different responses (*PP* and age) are depicted in Figure 6 (left and right columns for *PP* and age, respectively). We found that the first component (as well as the intercept term) was significantly predictive of both *PP* ($p<0.01$) and age ($p<0.05$).

**Figure 7:**
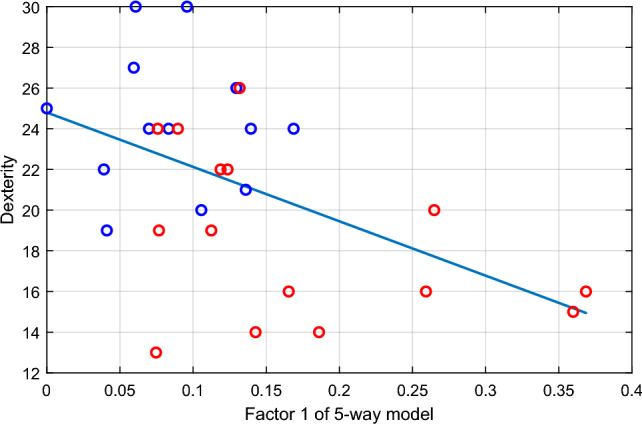
Correlation analysis results between factor 1 and PP (manual dexterity index) for all participants, except participant $p=29$ who is removed as an outlier. Dots represent different participants (blue for YA and red for OA, respectively) and the blue line is the linear regression line that fits the data. The computed correlation is $-0.50$ and the corresponding p-value is 0.007.

To further probe the (direction of) the relationship between the identified participant factors and age/manual dexterity, we first computed the correlation between the first component ($r=1$) and the dexterity vector *PP*. Having noticed that in the first participant component the entry of the last participant was much higher than other entries (as shown in Figure 4), we removed this potentially outlying value that could dominate the correlation outcome. We found a significantly negative correlation ($rho=-0.500, p=0.007$) indicating higher activation of the first factor for participants with low dexterity (Figure 7).

Overall, our findings show higher recruitment of the first component for participants with poor manual dexterity and advanced age.

In addition to manual dexterity, we have also investigated the correlation between the participant components and their tactile sensitivity. The latter was assessed using the Semmes-Weinstein monofilament test conducted for each participant ^64^; see Materials and Methods for further details. The result, however, shows that there is not a significant correlation between them, i.e., $p>0.2$ for all of the four participant factors.

## Discussion

In this study, we introduced a tensor decomposition and applied it to muscle activation and kinetic signals to extract motor patterns associated with object grasping and lifting and investigate any changes induced by ageing. This approach provided a novel methodology for the succinct characterisation of muscle couplings and related grip and load forces together with the corresponding temporal profiles during the lifting movements. Crucially, we extracted these patterns across participants, thus we could characterise differences in motor patterns attributed to ageing and/or motor decline.

Specifically, we identified three components (first, second and fourth) that activate the same set of muscles in different proportions thus producing different GF to LF ratios. These three components involve the ECR and APB muscles which are crucial for grasping and lifting objects. The first component activates ECR and APB synergistically with similar levels of activation and shows an increasing-first decreasing-later temporal profile, overall associated with higher GFs compared to LFs. This component is more prevalent in OAs compared to YAs and shows a positive correlation with age and a negative correlation with manual dexterity. The fourth component instead has a relatively higher activation of APB and a decreasing temporal profile over lifting time and is associated with a more economic application of GF relative to LF. This component is more prevalent in YAs compared to OAs. Finally, the second component also has a lower GF/LF ratio and is also used more by YAs. This component represents (decreasing over lifting time) activations of the APB muscles solely. Taken together, higher activation of ECR coupled with APB is associated with higher GF-LF ratios and is used more by OAs whereas higher activation of APB (coupled or uncoupled with ECR) is associated with lower GF-LF ratios and is used more by YAs. Thus, our findings align with the suggestion that ageing may be associated with a reduced capacity to individually activate the muscles in the forearm (ECR) and hand (APB), which may explain the less economic lifting strategy observed in OAs ^10^. This effect correlates strongly with a decrease in manual dexterity thus suggesting that the coupling between ECR and APB and higher GF/LF may be a compensatory mechanism for poor manual dexterity as a result of ageing ^21^. One could hypothesize that the higher GF-LF ratios observed are due to deteriorated haptic feedback of the manipulanda slipping through the hands (e.g., due to skin hardening or less tactile receptors), thereby forcing OAs to maintain a larger safety margin to avoid the object from falling. However, since tactile sensitivity scores from the Semmes-Weinstein monofilament test did not correlate with the obtained components, we are led to believe this hypothesis is not likely.

The presence of the same muscles in these three components also has implications about the effect of ageing on muscle synergies^42^. Our findings suggest that, although muscle recruitment remains intact with ageing, the balance of these muscle activations is altered thus leading to differential grip force generation. This may imply that ageing may not alter muscle recruitment but the relative composition of muscle synergies. In fact, this relative change may represent a new motor strategy adopted by older individuals to overcome the degradation in manual dexterity^21^.

The rationale for using different object loads was that these could have resulted in different grasping strategies depending on age, or, revealed age-dependent differences only for the high load as a result of increased task difficulty. The proposed approach could have captured these differences in the object load mode, via components that are activated only for one of the two objects, or, differentially between the two objects. By examining the age-dependent recruitment of these components, we could have identified interactions between load and age. However, our data did not reveal such an effect.

Concerning future use of the proposed approach, we suggest that the methodology developed here can be applied to a variety of motor behavioral experiments. Crucially, our approach enabled the joint analysis of EMG and kinetic measurements and consequently the identification of relationships between GF/LF ratios and muscle couplings which would not be directly observable with separate analyses of the two types of measurements (see also ^7,17,59^ for similar approaches in lower dimensions). Thus, this methodology will be useful when attempting to merge information and identify dependencies between different types of motor signals, such as neuromuscular, kinematic and kinetic recordings.

When the recorded motor signals contain responses across multiple locations (e.g., muscles or joints), times (e.g., different phases of movement execution), trials (repetitions of the same task), experimental conditions (e.g., varying distance, speed or load) and participants (with different age, gender and anthropometric measurements), they are naturally expressed as a 5-way tensor. The methodology we presented here decomposes such tensors into combinations of factors each of which has a spatial component (describing which muscles/forces are activated together), a temporal component (describing the temporal activation profile of the spatial pattern), a trial component (describing the level of recruitment of each spatiotemporal pattern in a given trial), a condition component (describing how much each pattern is used on each experimental condition – object weight here), and a participant component (describing the strength of each pattern activation for each participant). We also proposed a set of measures to evaluate the effectiveness of such a decomposition in both approximating the original recordings (VAF) and conveying information about differences across individuals (classification, correlation and regression analyses) ^15,19,30^. Interestingly, our approach also revealed that the level of activation of one component (the first) decreased across trials which led to relatively less use of this muscle and force pattern as the experiment progressed. Thus, the proposed methodology can be useful for such trial-by-trial analysis of motor pattern recruitment that may help quantify adaptation/learning or perhaps fatigue effects during the experiment. While a Mann-Kendall test was adopted here to reveal monotonic trial-by-trial trends, further assessment of motor control and learning will likely necessitate suitable exponential curve modeling of trial factors as well as protocol augmentation to include additional baseline and washout phases.

We contend that such a tensor decomposition can be particularly effective when the aim is to: a) tease apart motor patterns with different functional roles^18^; b) reveal their spatial and temporal representations^16^; c) quantify their relative contribution to discrimination between experimental conditions^19,20^; d) identify trial-by-trial differences in their recruitment and any resulting trends; and e) assess how these patterns may differ across individuals or populations with common characteristics. The latter was the main aim of this study which revealed muscle couplings and corresponding forces that predict age and/or manual dexterity differences across participants. In future work, differences in tensor structure can be used to test specific hypotheses about how motor signals differ between healthy and impaired individuals (e.g., stroke or spinal cord injury patients) and potentially inform rehabilitation or treatment strategies^31,48,53^. Moreover, our approach relies on pooling all subjects together to obtain the participant factor which is used to predict age and dexterity. From a clinical perspective, a method that operates on a subject-by-subject basis to generate evaluations would also be of interest. While we do not pursue this direction here, our approach could accommodate this use case by projecting an unseen subject’s data onto the extracted factors to obtain a new score in the participant vector. This score can be subsequently used to predict the subject’s age or manual dexterity. However, to make this generalizable, a larger population size will be required to validate the obtained results.

Going back to the data analysis methodology, the CANDECOMP/PARAFAC decomposition is one of the most widespread methods for low-rank tensor factorization, which is unique under very mild conditions ^9^. Indeed, Harshman introduced PARAFAC in 1970 because it eliminates the ambiguity associated with two-dimensional principal components analysis (PCA), and thus has better uniqueness properties ^37^. When non-negativity constraints are incorporated, the NCP can also be viewed as a high-order extension of the non-negative matrix factorization (NMF)^38^ algorithm typically used to identify muscle synergies from EMG data^2,13,60^. While one could always unfold (or matricize) a tensor to obtain a matrix and resort to classical data analysis methods such as PCA or NMF, this can be undesirable for several well-documented reasons ^9^. Interpretability (due to uniqueness) of the decomposition outputs^8,9^ and the lack of any other assumptions, such as orthogonality, enable the extraction of non-orthogonal motor patterns, which are often sparse but partly overlapping, such as the ones typically generated by neural circuits with hard-wired connectivity^14,45,65^. Furthermore, the NCP decomposition has only one free parameter, the rank of the tensor *R*, i.e., the number of outer-product components that are sufficient to describe the input data^37^. Relative to matrix-based NMF approaches that extract muscle synergies from 2-way (space-time) EMG signals, a tensor decomposition approach offers a richer, multi-view analysis that can also facilitate identification of individual participant and trial patterns. Together, these properties make the NCP decomposition an attractive and natural method for identifying low-dimensional representations of muscle activations and resulting forces that may differ across time, repetitions, experimental conditions and/or individuals.

Tradeoffs arise and our experimental design and methodology are not devoid of limitations. We comment on three important ones here. First, here we estimated load force using an accelerometer and did not directly measure it using a uni-directional load cell that can measure its vertical component. A limitation of our approach is that it does not allow estimation of LF during the load phase, i.e., before the object is lifted from the table. However, once the object is airborne it provides a more complete picture of LF, capturing both its vertical and sagittal components (the latter was negligible here). Second, we normalized EMG data to their maximum amplitude across trials for each participant. This way, recordings have similar scales across individuals. Since the tensor decomposition algorithm extracts components that explain as much variability in the data as possible, this normalization ensures that the extracted factors will not be dominated by a subset of participants just because their EMG amplitudes are markedly higher. Our subsequent analyses further showed that the extracted components can discriminate between OAs and YAs, which (arguably) serve as a form of validation to the adopted normalization in this context. However, the price paid is that EMG amplitudes per se are no longer comparable across participants; see also ^6^ for a detailed treatment on the challenging issue of EMG amplitude normalization. Lastly, we comment on the choice of the classification algorithm. While admittedly very powerful, artificial neural networks and related (complex) deep learning models often require large datasets to attain satisfactory classification performance. Given our limited sample size and the multi-way nature of our data, here we opted for an NCP-based feature extractor followed by a simple linear support vector machine (SVM) classifier.

## Material and methods

### Participants

Human participants of two different age groups took part in the study, 13 YA (<30 years old, three left-handed, M = 22.2 ± 2.59 yrs old; F = 11) and 16 OA (>60 years old, two left-handed, M =70.8 ± 7.42 yrs old; F = 8). Hand dominance was self reported. All participants ($P=29$) had no known musculoskeletal or neurological conditions and normal or corrected vision. This research was approved by the Research Ethics Committee of the Faculty of Biological Sciences of University of Leeds and all methods conformed to the Declaration of Helsinki and were carried out in accordance with the University’s regulations. Written informed consent was obtained by all participants following guidelines of the University of Leeds.

### Clinical tests

Before the experimental session, we performed two clinical tests to assess the participants’ tactile sensitivity and manual dexterity. We used the Semmes-Weinstein (SW) monofilament test^64^ as a measure of cutaneous sensitivity. The testing kit includes several monofilaments of varying thickness so that each monofilament flexes under a specific force. We tested eight sites (four per hand) on the participants’ hands; namely, middle fingertip, index fingertip and thumb tip. For each test site, and starting from the finest monofilament, the monofilament was pressed into the skin until the filament lightly flexed. Participants were asked if they felt the touching sensation (if the answer was negative, the next finest monofilament was used), and the target force required to elicit a response was recorded for each site. The sum force was calculated for each hand and each participant as a SW score of tactile sensitivity. We also quantified manual dexterity using the Purdue Pegboard (PP) test, which includes sub-tests for assessing dominant, non-dominant and bimanual levels of dexterity, with all sub-levels of the test providing high levels of reliability (r = 0.60 to 0.86) – specifically when working with OA (r = 0.66 to 0.90) ^22^. The test requires the participant to see how many pins they can place into the allocated holes within a 30-second time period. Participants performed the test bimanually to obtain an overall measure of their manual dexterity.

### Apparatus

To perform the grasping tasks, we built two manipulanda made from carbon-filled nylon (width: 40 mm, height: 110 mm, depth: 50 mm) and containing 50 N load cells (Omega, LCM201-50) which enabled recording grip forces (GF); see also Figure 2 (A). GF data were acquired using a 16-bit data acquisition card (National Instruments, USB-6002) and processed using a custom-built program in Labview (v.14). Reliability of recordings was ensured by prior validation tests ($< 1\%$ error for forces between 1 and 36N). To track the object kinematics (200Hz sampling frequency), we attached four Qualisys markers to each manipulandum and used Qualisys camera setups (12 cameras in the lab, 5 in the community centres). For both setups, calibration was successful according to guidelines (error $< 1.0$mm).

### Grasping and lifting task

All participants sat on a chair in front of a table^34^; see also Figure 2. The table surface was level with their navel and their feet were flat on the ground. For each participant the grasping task was normalised by placing the manipulanda 75% of shoulder width and 70% of maximum reach, as previous research has shown gross changes in shoulder and elbow flexion/extension can affect grip force and load force coordination during lifting ^61^. Shoulder width was measured as the distance between the acromion process, and reach was measured from the acromion process to the tip of the index finger with the participants sat with their dominant arm extended onto the table. The participants’ fingers and thumbs were cleaned with alcohol wipes. The researcher demonstrated how to pick up the manipulanda using the two circular plungers, with a precision grip. Participant instructions were to “grasp the objects and lift them level with a target height placed in front of them (300mm height), and to hold the objects as still as possible”^34^. After a 10-second period, the researcher asked the participants to replace the object(s) back on the starting markers. Participants performed $K=10$ consecutive repetitions of bimanual grasp-lift-replace movements with $J=2$ object masses: light (200g) and heavy (400g). These repeated tasks were performed in a blocked, randomised order to mitigate for any learning effect.

### EMG data collection

EMG data were collected from the Anterior Deltoid, Flexor Carpi Radialis (FCR), Extensor Carpi Radialis (ECR) and Abductor Pollicis Brevis (APB) of both arms and hands using eight Delsys Trigno™ sensors (sampling frequency of 2,000Hz). Given the nature of the grasping and lifting task, the intention was to collect detailed forearm and hand data. However, the ability to get reliable measurements via surface EMG was limited due to the size of the sensors compared to the atrophy within the aged population. For this reason, sensors were placed on muscles where EMG signals could be consistently and reliably collected given the population. We thus chose a prime mover for shoulder flexion (Anterior Deltoid), two wrist stabilisers (ECR and FCR), and a thumb abductor (APB).

### Data pre-processing

Six degree-of-freedom models were created in Qualisys for each object and were used to compute the position (*x*, *y*, *z*), velocity and acceleration of each object. Load force was calculated from the objects’ mass times a product of the acceleration (vertical and sagittal) plus gravity^28,29,44^ as follows:

1 $$\begin{aligned} LF = m \times \sqrt{(a_z + g)^2 + a_y^2}, \end{aligned}$$

where *m* is the object mass (0.2 or 0.4 kg), $a_z$ is the vertical acceleration, $a_y$ is the acceleration in a sagittal plane, and *g* represents gravity (9.81 $m/s^2$). Grip force (GF) and load force (LF) data were then filtered using a 4th order low-pass Butterworth filter with a 12Hz cut-off. Based on the design of our manipulanda to measure GF, LF cannot be calculated when the object is in contact with the table, which meant we had to group the pre-loading (GF started but no LF) and loading (GF applied and LF < object weight) into one phase. Despite missing out on more detailed pre-loading and loading data, the advantage of not including an LF cell is that it allows a far larger range of movement, better-representing participants picking up objects in everyday situations. Crucially, once the object is airborne the measurement of LF via this approach has been validated in previous studies; see e.g., ^25,28^. The small caveat in validity with our and previous researchers’ approach ^25,28^ is if the object is not lifted in the vertical plane (excessive movements in the sagittal/frontal plane). We emphasised this vertical displacement to our participants and checked it thoroughly in our data to ensure there were no such issues with the calculation of LF. Finally, to obtain smooth force profiles, GF and LF recordings were low-pass filtered (12Hz cut-off, 4th-order Butterworth).

The EMG recordings for each trial were digitally, full-wave rectified, low-pass filtered (10Hz cut-off, 4th order Butterworth, zero-phase distortion; R Signals package, filtfilt function) and down-sampled to 200Hz to align with kinetic and kinematic datapoints. All trials were visually scanned for artifacts and affected trials were excluded from further analysis ($<5\%$ of total number of trials)^32^.

For each trial, EMG, GF and LF data were selected during the dynamic phase of object lifting - from the point of first contact to the end of the object lift / beginning of the stable phase of the object hold. Determination of this time window was done based on the recorded kinematic data of the object position. Specifically, first contact was defined as when the grip force exceeded 0.1N, while the stable phase began when velocity returned to < 0.001m/s after object lift. To account for electromechanical delays between EMGs/forces and resulting kinematics, we selected EMG, GF and LF measurements starting 100ms before the first contact with the object and ending 100ms after beginning of the stable phase of object hold.

EMG signals of all muscles, GFs and LFs were time-normalised to $T_0=500$ datapoints to ensure equal temporal weighting across participants and conditions for the subsequent analysis. Then, EMG data and bimanual GFs and LFs for each trial ($K=10$ for each object *J*) of each participant were concatenated to form a $12\times 500$ matrix containing all recordings from 8 muscles and 4 forces ($M=12$) over $T_0=500$ datapoints. Finally, to remove edge artifacts from signal filtering, the first and last 20 samples were removed from each recording, which resulted in signals of $T=460$ datapoints.

### Tensor formulation and decomposition

The aforementioned data was collated across objects (*J*), trials (*K*), and participants (*P*, ordered according to age from youngest to oldest) to construct the 5-way tensor $\underline{{\textbf{X}}}^{(5)}\in \mathbb {R}_+^{M\times T\times J\times P\times K}$, where $M=12$, $T=460$, $J=2$, $P=29$, and $K=10$.

**Normalization of concatenated muscle and force recordings**. When concatenating different types of signals as inputs to a tensor decomposition (here the EMG data and bimanual GFs and LFs a described in the Data pre-processing section), it is possible that a subset of these signals may be ignored as a result of differences in scale; i.e., lower values may “swamped” in the components as they account for less variance in the data. In our case, we noted that the overall amplitude of the force signal ($m=1,...,4$) was much less than the amplitude of the EMG signals ($m=5,...,12$). Under the consideration that we wanted to balance the contribution of both of the force signal and EMG in the following analysis but the relative signal strength within each spatial group (force or EMG) should not be disturbed, the force signals and EMG were normalized so that both have the same mean $\ell _2$-norm (which can be interpreted as the energy of the signals). As an example, if we rearranged the 5-way tensor $\underline{{\textbf{X}}}^{(5)}$ as a matrix ${\textbf{X}}_M\in ^{M\times KTJP}$, then the sub-matrix$[{\textbf{X}}_M]_{1:4,\cdot }$ (containing the first four rows of ${\textbf{X}}_M$) was re-scaled so that the mean $\ell _2$ norm of the first four rows (the force signals) and the last eight rows (the EMG signals) of ${\textbf{X}}_M$ would be equal. With this normalization, we found the magnitude of the estimated force and muscle factors were at the same level and comparable.

**Non-negative canonical polyadic (NCP) decomposition.** We applied the NCP decomposition to the 5-way EMG data data tensor $\underline{{\textbf{X}}}^{(5)}\in \mathbb {R}_+^{M\times T\times J\times P \times K}$. NCP entails a low-rank approximation $\underline{{\textbf{X}}}\simeq {\textbf{A}}\circ {\textbf{B}}\circ {\textbf{C}}\circ {\textbf{D}}\circ {\textbf{E}}$ subject to non-negativity constraints on the entries of the so-termed factor matrices $\{{\textbf{A}},{\textbf{B}},{\textbf{C}},{\textbf{D}},{\textbf{E}}\}$. Non-negativity is well motivated to better interpret the EMG and net load force data. The outer product ($\circ$) decomposition is defined such that scalar tensor entries $X_{mtjpk}$ are approximated as

2 $$\begin{aligned} \begin{aligned} X_{mtjpk}^{(5)}&\simeq \sum \limits _{r = 1}^R A_{mr} B_{tr} C_{jr} D_{pr} E_{kr}, \end{aligned} \end{aligned}$$

where *R* is the tensor rank adopted for the approximation. Matrix ${\textbf{A}}\in \mathbb {R}_+^{M\times R}$ denotes the spatial (force and muscle) factor and its columns $\{{\textbf{a}}_r\}_{r=1}^R$ represent the spatial synergies. The row indexing of forces and muscles follows the description in Table 1. Likewise, matrix ${\textbf{B}}\in \mathbb {R}_+^{T\times R}$ is the temporal factor and its columns $\{{\textbf{b}}_r\}_{r=1}^R$ are the temporal activation sequences of each component. Matrices ${\textbf{C}}\in \mathbb {R}_+^{J\times R}$ and ${\textbf{D}}\in \mathbb {R}_+^{P\times R}$ are the object and participant factors, respectively, that capture the object-wise and participant-wise information in the data. Finally, ${\textbf{E}}\in \mathbb {R}_+^{K\times R}$ is the trial factor.

Given $\underline{{\textbf{X}}}^{(5)}$ and a prescribed value of *R* (the method used to choose *R* is described below), the factor matrices are estimated by solving the following non-convex NCP decomposition problem

3 $$\begin{aligned} \min _{\{{\textbf{A}},{\textbf{B}},{\textbf{C}},{\textbf{D}},{\textbf{E}}\} \in \varvec{\Omega }}\,\sum _{mtjpk}\left( X_{mtjpk}^{(5)} - \sum \limits _{r = 1}^R A_{mr} B_{tr} C_{jr} D_{pr} E_{kr}\right) ^2, \end{aligned}$$

where $\varvec{\Omega }$ is the feasible set imposing all optimization variables (i.e., the entries of the factor matrices $\{{\textbf{A}},{\textbf{B}},{\textbf{C}},{\textbf{D}},{\textbf{E}}\}$) are non-negative. The optimization problem (3) was solved via the *structured tensor fusion* (SDF) algorithm ^56^, specifically by using the sdf_nls function from Matlab’s toolbox Tensorlab ^62^.

Multi-way decompositions such as NCPD suffer from an inherent scaling ambiguity. In order to fix the scale, the estimated factors were normalized by letting $\Vert {\textbf{A}}\Vert _F =\Vert {\textbf{B}}\Vert _F = \Vert {\textbf{D}}\Vert _F = \Vert {\textbf{E}}\Vert _F = 1$ and ${\textbf{C}}\leftarrow \Vert {\textbf{A}}\Vert _F\Vert {\textbf{B}}\Vert _F\Vert {\textbf{D}}\Vert _F\Vert {\textbf{E}}\Vert _F{\textbf{C}}$, where $\Vert \cdot \Vert _F$ denotes the Frobenius norm of its matrix argument. For $R=4$ components, the estimated factors are depicted in Figure 4.

**Figure 8:**
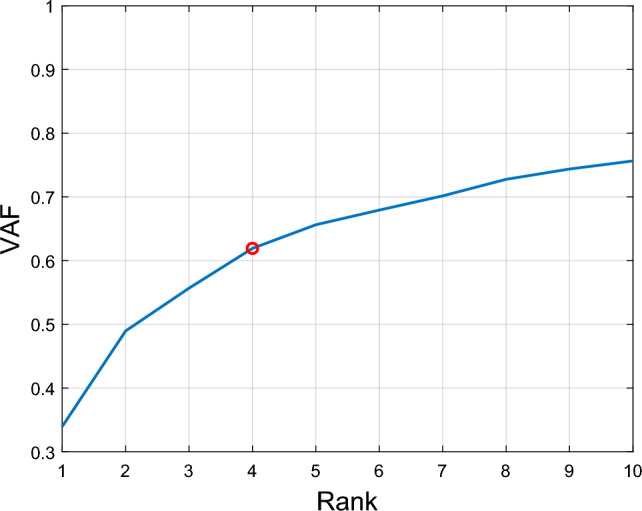
Rank determination for the 5-way tensor. The blue curve shows the average VAF (10 realizations for each data point), obtained for each value of the rank ranging from $R=1$ up to 10 factors. With $R=4$ factors, the obtained NCP decomposition of the 5-way data tensor accounts for more than $60\%$ of the variance in the recordings.

**Variance accounted for (VAF) and rank selection**. The sole parameter for the NCP decomposition is the rank *R* of the tensor approximant, which naturally affects model complexity as well as the reconstruction error. In order to assess how well the multi-way signal is reconstructed from the estimated synergies, the variance explained by the NCP decomposition is evaluated for different values of *R*. Following a well established criterion, see e.g., ^27^, the smallest value of *R* is chosen that explains at least $60\%$ of the variance in the original data tensor. To this end, the Variance Accounted For (VAF) metric defined as

4 $$\begin{aligned} \begin{aligned} \text {VAF}&= 1 - \frac{\text {MSE}}{\text {var}(\text {vec}(\underline{{\textbf{X}}}^{(5)}))} \end{aligned} \end{aligned}$$

was evaluated, where the mean squared error (MSE) was computed as $\text {MSE} = \Vert \text {vec}(\hat{\underline{{\textbf{X}}}}^{(5)}) - \text {vec} (\underline{{\textbf{X}}}^{(5)})\Vert ^2/(MTJPK)$ and $\hat{\underline{{\textbf{X}}}}^{(5)}$ represents the reconstructed tensor. In other words, the MSE corresponds to the cost function in the NCP optimization problem (3), up to scaling

The VAF was computer over a grid of candidate values $R = 1,\ldots ,10$. Results are depicted in Figure 8. With $R=4$ factors, the obtained NCP decomposition of the 5-way data tensor satisfies the target criterion. Besides, $R=4$ was also the second largest change of VAF slope, while the largest change of VAF slope was at $R=2$ and trivial.

**Mann-Kendall test for the trial factor**. We applied the Mann-Kendall test to the estimated trial factors $\{{\textbf{e}}_r\}_{r=1}^R$ to examine whether there was a significantly increasing or decreasing trend over trials from $k=1$ to 10, with the significance level set at $\alpha = 0.05$.

### Correlation analyses

Correlation analyses were conducted to examine if there is any significant relationship between the recruitment of the estimated muscle/force couplings and clinical measures such as manual dexterity and tactile sensitivity, or participant characteristics (age). Specifically, the correlation was computed between the participant mode factors of the identified decomposition (i.e., the columns $\{{\textbf{d}}_{r}\}_{r=1}^R$ of matrix ${\textbf{D}}\in \mathbb {R}_+^{P\times R}$) and the $P\times 1$ vectors of manual dexterity (*PP*) and age information collected across all participants. Bonferroni correction for multiple comparisons was implemented.

### Classifying age groups via linear support vector machine (SVM)

An age classification analysis was conducted to identify any differences in muscle recruitment and GF/LF relationship between the two age groups. Specifically, the columns $\{{\textbf{d}}_{r}\}_{r=1}^R$ of the participant factor matrix ${\textbf{D}}\in \mathbb {R}_+^{P\times R}$ (representing the level of recruitment of each factor for each participant) were used as predictors of the participant age group (YA vs. OA). To this end, a linear support vector machine (SVM) classifier was adopted and trained using leave-one-out cross-validation, which is appropriate when the sample size is limited. Matlab’s Classification Learner app was used for the implementation.

An iterative procedure was utilized in order to identify the participant factors (and combination thereof) that were most discriminative of age. First, each individual participant factor was separately used as predictor of age group. This served to determine the most discriminative factors. The predictive power of pairs of factors was subsequently examined (to assess if their combination has more age classification power), then triplets, and so forth. Classification accuracy (% of correctly classified participants) and area and the ROC curve (AUC) were adopted as measures of classification performance.

A permutation test was also conducted to assess if inclusion of more factors significantly improves classification performance. Specifically, to assess significance for factor *r*, the activations of this component were randomly permuted across participants 100 times, and empirical distributions of classification accuracy and AUC values across the 100 permutations were obtained. This serves to randomize the contribution of factor *r* to classification performance, while keeping the contributions of the other factors used in the classifier intact. The percentile above which the actual (unpermuted) classification performance lies was calculated and this whole process was repeated for each of the other ($\ne r$) factors fed to specific classification rule under study.

### Predicting age and manual dexterity via linear regression

Linear regression was utilized to further assess how well manual dexterity and age can be predicted by combinations of the identified factors. The participant factors ${\textbf{D}}\in \mathbb {R}_+^{P\times R}$ served as predictors and the $P\times 1$ age vector ${\textbf{y}}^{(\text {age})}$ (or the manual dexterity vector ${\textbf{y}}^{(PP)}$) as response variables. The linear regression model $y^{(\text {age})}_p = \bar{{\textbf{d}}}_{p}{\textbf{b}}+ b_0+\epsilon _p,\,p=1,\ldots ,P,$ was implemented, where $\bar{{\textbf{d}}}_{p}\in \mathbb {R}_+^{1\times R}$ denotes the *p*-th row of ${\textbf{D}}$ and $\epsilon _p$ is a zero-mean noise. Regression coefficients ${\textbf{b}}\in \mathbb {R}^{R}$ (and the corresponding p-values) were estimated using the fitlm function in Matlab, and for each predictor these coefficients represent the age- or dexterity-predictive power of each factor. The linear regression results are shown in Figure 6.

## Data Availability

The datasets used and analysed during the current study are available from the corresponding author on reasonable request.
